# Sexually dimorphic neuroanatomical differences relate to ASD-relevant behavioral outcomes in a maternal autoantibody mouse model

**DOI:** 10.1038/s41380-021-01215-w

**Published:** 2021-07-21

**Authors:** Matthew R. Bruce, Karen L. Jones, Anthony C. Vernon, Jill L. Silverman, Jacqueline N. Crawley, Jacob Ellegood, Jason P. Lerch, Judy Van de Water

**Affiliations:** 1 Department of Internal Medicine, Division of Rheumatology, Allergy, and Clinical Immunology, University of California, Davis, CA USA; 2 MIND Institute, University of California, Davis, CA USA; 3 Department of Basic and Clinical Neuroscience, Institute of Psychiatry, Psychology and Neuroscience, King’s College London, London, UK; 4 MRC Centre for Neurodevelopmental Disorders, King’s College London, London, UK; 5 Department of Psychiatry and Behavioral Sciences, University of California, Davis, CA USA; 6 Mouse Imaging Centre (MICe), Hospital for Sick Children, Toronto, ON Canada; 7 Department of Medical Biophysics, The University of Toronto, Toronto, ON Canada; 8 Wellcome Centre for Integrative Neuroimaging, The University of Oxford, Oxford, UK

**Keywords:** Autism spectrum disorders, Biological techniques

## Abstract

Immunoglobulin G (IgG) autoantibodies reactive to fetal brain proteins in mothers of children with ASD have been described by several groups. To understand their pathologic significance, we developed a mouse model of maternal autoantibody related ASD (MAR-ASD) utilizing the peptide epitopes from human autoantibody reactivity patterns. Male and female offspring prenatally exposed to the salient maternal autoantibodies displayed robust deficits in social interactions and increased repetitive self-grooming behaviors as juveniles and adults. In the present study, neuroanatomical differences in adult MAR-ASD and control offspring were assessed via high-resolution ex vivo magnetic resonance imaging (MRI) at 6 months of age. Of interest, MAR-ASD mice displayed significantly larger total brain volume and of the 159 regions examined, 31 were found to differ significantly in absolute volume (mm^3^) at an FDR of <5%. Specifically, the absolute volumes of several white matter tracts, cortical regions, and basal nuclei structures were significantly increased in MAR-ASD animals. These phenomena were largely driven by female MAR-ASD offspring, as no significant differences were seen with either absolute or relative regional volume in male MAR-ASD mice. However, structural covariance analysis suggests network-level desynchronization in brain volume in both male and female MAR-ASD mice. Additionally, preliminary correlational analysis with behavioral data relates that volumetric increases in numerous brain regions of MAR-ASD mice were correlated with social interaction and repetitive self-grooming behaviors in a sex-specific manner. These results demonstrate significant sex-specific effects in brain size, regional relationships, and behavior for offspring prenatally exposed to MAR-ASD autoantibodies relative to controls.

## Introduction

Autism spectrum disorder (ASD) is a set of heterogeneous neurodevelopmental disorders that are behaviorally classified by socio-communicative impairments accompanied by the presence of repetitive and restrictive interests and behaviors [1]. One potential non-genetic contributor to ASD is immune dysregulation, which has been described in individuals with ASD and their family members [2]. Most notably, some mothers of children with ASD have been reported to have circulating autoantibodies reactive to fetal brain proteins [3, 4] (reviewed in [5]).

Our lab has identified eight protein antigens for maternal autoantibody related (MAR) risk of ASD: lactate dehydrogenase A and B, stress-induced phosphoprotein 1, collapsin response mediator proteins 1 and 2, guanine deaminase, Y-box binding protein 1 [6], and neuron-specific enolase [7]. In addition, we mapped the antigenic epitope sequences for each of the proteins recognized by these ASD-specific maternal autoantibodies [8]. Then we created an antigen-driven mouse model for MAR risk of ASD in which autoantibodies reactive to the salient epitope sequences are generated in female dams prior to breeding. In this model, male and female offspring prenatally exposed to the maternal autoantibodies had significant alterations in developmental milestones, reduced social interactions during dyadic play, and exhibited increases in repetitive self-grooming behaviors [9]. However, there remains a critical need to identify the underlying biological mechanisms that lead to MAR-ASD.

In the current study, we examined the potential effects of brain-reactive maternal autoantibodies on neuroanatomy through cross-sectional analysis of offspring at 6 months of age. To accomplish this we conducted high-resolution ex vivo magnetic resonance imaging (MRI) on adult MAR-ASD and control offspring that had undergone behavioral testing in our previously published study [9]. In this manner, we were able to perform direct correlational analysis between regional brain volume and behavioral outcomes to provide a comprehensive readout of potential pathology. Additionally, we used structural covariance analysis to interpret network-level dysregulation of brain volume in response to MAR-ASD autoantibody exposure.

## Methods

### Animals

MAR-ASD and control mice were previously created and studied in the Van de Water Lab [9]. A total of *n* = 22 MAR-ASD mice (11 male, 11 female) and *n* = 23 control mice (12 male, 11 female) aged approximately six months were perfused for MRI imaging. Please see supplemental methods for additional animal information.

### Magnetic resonance imaging

A multi-channel 7.0 Tesla (7.0-T) MRI scanner (Agilent Inc., Palo Alto, CA) was used to image the brains within the skulls. A custom-built solenoid coil array was used to image 16 brains in parallel [10]. Parameters for the ex vivo MRI scans were as follows: T2-weighted, 3-D fast spin-echo sequence, with a cylindrical acquisition of k-space, and with a TR of 350 ms, and TEs of 12 ms per echo for six echoes, field-of-view of 20 × 20 × 25 mm^3^ and matrix size = 504 × 504 × 630 giving an image with 0.040 mm isotropic voxels. Total imaging time for the acquisition was 14 h [11]. For details on registration and analysis please see Supplementary methods.

### Structural covariance

To assess structural covariance by region within the dataset, the absolute volumes of all 159 atlas-segmented regions were subjected to correlational analysis using Pearson’s *r* as a readout. To reduce the number of comparisons for subsequent statistical analyses correlational data were then grouped into six clusters (Posterior Cortical, Hippocampal, Anterior Cortical, Subcortical, Midbrain, and Brainstem, and Cerebellum) defined previously by others using hierarchal clustering of structural covariance in the mouse brain [12]. The identity of regions assigned to each cluster is detailed in Supplementary Table 2. Following cluster assignment, correlation values for each brain region within a cluster were then averaged, similar to that described previously [13]. Mean correlation values were then compared between treatment conditions and sex using Kruskal-Wallis non-parametric testing, with corrections for multiple comparisons using a two-stage step-up FDR method at a level of 5%. All data analysis for these methods was performed using Prism 8 with visualization conducted in R.

### Behavioral correlations

To identify potential relationships between offspring behavior and absolute regional brain volume, an exploratory analysis was performed to correlate behavioral data with MRI-based neuroanatomical findings. In particular, volumes were correlated with the following behaviors previously collected by our group [9]: juvenile reciprocal social interactions (JRSI), self-grooming in an empty cage, and male-female social interaction (MFSI) behaviors. Neuroanatomical regions correlated with behavioral findings were limited to areas identified passing 5% FDR correction in the Full and female-only groups. Relationships were assessed via Pearson’s correlation. Pearson’s correlations were performed using SPSS software (SPSS Version 25.0; IBM Corp., Armonk, NY); *p* values < 0.05 for two-tailed tests were considered to be statistically significant. As these correlations were exploratory, no corrections for multiple comparisons were made. Data visualization and clustering were conducted using the online tool ClustVis (https://biit.cs.ut.ee/clustvis/).

## Results

### MAR-ASD offspring display sex-specific increases in brain volume

**Fig. 1:**
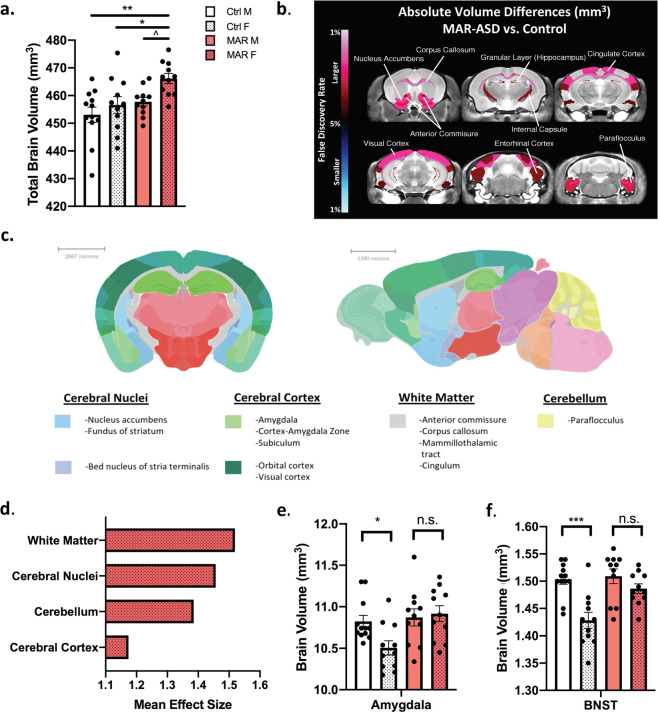
Female MAR-ASD offspring exhibit increases in total and regional brain volume. **a** Differences in total brain volume between treatment and sex. **b** A false discovery ratio (FDR) heatmap of significant regional differences between MAR-ASD and control animals, shown combined between sexes (Full Group). Anything highlighted in red is significantly larger in MAR-ASD compared to control animals and anything blue is significantly smaller at an FDR value of <5%. **c** Coronal and sagittal images of the mouse brain overlaid with colorimetric classification of brain areas as defined by the Allen Mouse Brain Atlas. Headings under brain sections denote areas where significant regional volumetric differences were seen in female MAR-ASD mice. **d** Average effect sizes among brain areas identified to exhibit regional changes. Data are expressed as Cohen’s d values. **e**, **f** Volumetric differences in sexually dimorphic brain regions compared within treatment conditions between sexes in the amygdala (**e**) and bed nucleus of stria terminalis (BNST) (**f**). Statistical analyses were conducted using a one-way ANOVA. For data included in figures **a** and **d**–**f**: MAR (N = 11M, 11F), Ctrl (N = 12M, 11F). Error bars represent mean ± SEM. * = *p* < 0.05, ** = *p* < 0.01, *** = *p* < 0.001, ^ = 0.1 > *p* > 0.05, n.s. = non-significant.

Analysis of ex-vivo structural MRI data revealed MAR-ASD treatment-induced differences in total and regional brain volume at 6 months of age. Overall, female MAR-ASD mice exhibited significantly larger total brain volume (TBV) relative to both male and female control animals (male, *p* < 0.01; female *p* < 0.05), with a trending difference in TBV noted between sexes in MAR-ASD mice (Fig. 1a). No differences were observed in male MAR-ASD animals compared to controls. To evaluate differences by brain region, volumetric analysis of 159 separate regions was conducted. Analysis revealed that 20% (31/159) of regions examined were found to differ significantly in absolute volume (mm^3^) when comparing the MAR-ASD Full Group, including both sexes, to controls at an FDR of less than 5% (Fig. 1b; Supplementary Table 1). Assessment of regional volumetric differences in MAR-ASD mice split by sex revealed that 12% (20/159) of brain regions examined displayed significant increases in absolute volume in females, at an FDR of <5%. No statistically significant differences were observed in males (Supplementary Table 1). Additionally, no differences were observed in relative regional volumes for MAR-ASD mice compared to control animals for either sex. Collectively, these data suggest that female MAR-ASD mice were primarily driving the neuroanatomical phenotype seen in the Full Group comparison.

Manual annotation of the 20 regions displaying significant (5% FDR) increases in female MAR-ASD mice, using data from the Allen Mouse Brain Atlas, revealed that volumetric increases in female MAR-ASD mice were predominately seen in 4 brain areas: the cerebral nuclei, cerebral cortex, white matter, and the cerebellum. Specific regions affected are listed under each respective area (Fig. 1c) and included with more detail in the supplementary data (Supplementary Table 1). To investigate the magnitude of these volumetric changes, regional effect sizes were calculated across the MAR-ASD Full Group, and both sexes independently using Cohen’s d as a metric. Effect size averaging among regions within the affected brain areas revealed white matter regions to be the most prominently affected (Fig. 1d). While analysis by region showed that the largest volumetric differences were observed in the anterior commissure, medial orbital cortex (mOFC), and nucleus accumbens (NAc) in female MAR-ASD mice (Supplementary Fig. 1a). Using data provided by the Allen Mouse Brain Connectivity Atlas (http://connectivity.brain-map.org/), an exploration of projections from the mOFC revealed that main efferent projections pass through the caudoputamen and NAc, centered around the anterior commissure pars anterior (Supplementary Fig. 1b). These findings suggest that MAR-ASD exposure results in sex-specific regional volumetric differences within the brains of offspring that may involve altered cortico-striatal connectivity.

### MAR autoantibody exposure results in masculinization of sexually-dimorphic brain areas in female mice

Given that the volumetric increases in regional brain size of MAR-ASD animals were driven primarily by females, and the fact that multiple sexually dimorphic regions in the brain were among those found to be significantly enlarged in response to MAR-ASD exposure, (specifically, the amygdala and bed nucleus of stria terminalis (BNST) (Female *q* values = 0.04)), we investigated the possibility that MAR-ASD treatment may result in changes in the sexual dimorphism of these regions. To examine this, we compared the volume of specific brain regions, the amygdala, BNST, and the hypothalamus between MAR-ASD and control mice. These were selected a priori based on evidence in the literature confirming sexual dimorphism and association with ASD [14, 15]. As expected, analysis of regional brain volume within these areas in control mice corroborated the sex differences reported in the literature, with female control animals displaying significantly lower amygdala (*p* < 0.01; Fig. 1e) and BNST (*p* < 0.001; Fig. 1f) volume compared to male controls. The size of the hypothalamus also appeared lower in female control animals compared to males, but differences did not reach statistical significance (*p* = 0.09; Supplementary Fig. 2a). However, when assessing sex differences in these same regions in MAR-ASD mice, brain size in females was no different than that of males across each of the sexually dimorphic regions examined (Fig. 1e, f; Supplementary Fig. 2a). Together these findings relate a loss of sexual dimorphism in amygdala and BNST volume in response to gestational MAR-ASD exposure in mice, representing “masculinization” of these regions in females.

### Structural covariance analysis reveals desynchronized regional development in MAR-ASD offspring

Interestingly, while regional MRI analysis uncovered female-specific increases in brain size in MAR-ASD offspring, prior data collected on these same animals related that treatment-induced behavioral abnormalities affected male and female mice similarly [9]. A plausible explanation for this could be that MAR-ASD treatment results in network level changes in the brain volumes that are not apparent when assessing regions individually. Previous work has found that the volume of distinct neuroanatomical systems is tightly correlated, forming structural covariance networks in the brain in both humans and rodents [12, 16]. In addition, recent data suggest that these networks may be sensitive to immune challenge during neurodevelopment [17]. Therefore, we used structural covariance analysis to examine correlations between clusters of brain regions to provide a broader picture of neuroanatomical changes in response to MAR-ASD autoantibody exposure.

**Fig. 2:**
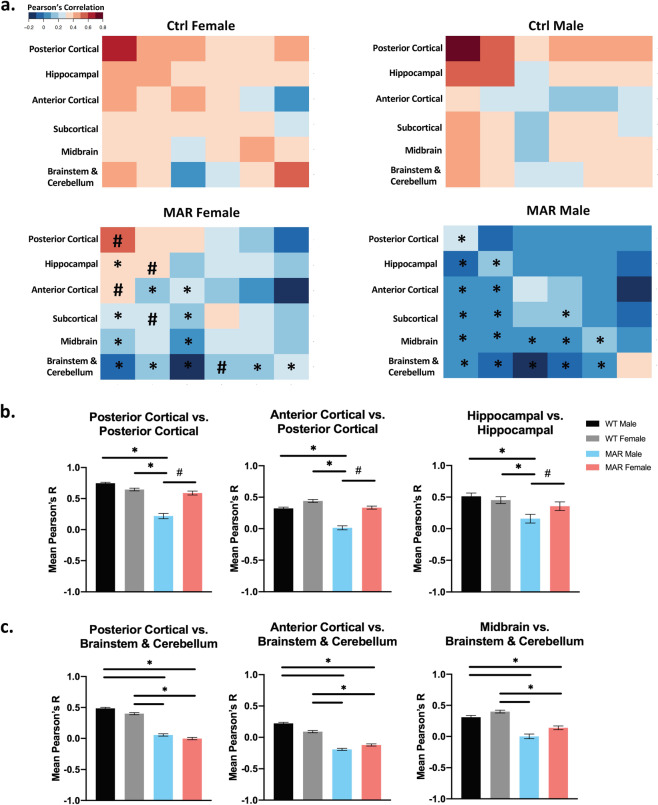
Analysis of regional structural covariance in the brains of MAR-ASD and control mice. **a** Heatmap-based visualization of regional correlation values organized into clusters based on anatomical location. Data represented as mean Pearson’s *r* values with results split by treatment and sex. **b** Plots displaying averaged correlation values across brain region clusters determined to be different in male MAR-ASD mice only. **c** Plots displaying regional differences as a result of treatment. Data for bar plots correspond to mean Pearson’s *r* values derived from correlational cluster analysis of animals within a treatment group (MAR (*N* = 11M, 11F), Ctrl (*N* = 12M, 11F)). Final *N*’s for statistical analysis reflect multiplication between regions within respective clusters (posterior cortical (*N* = 19), hippocampal (*N* = 13), anterior cortical (*N* = 24), subcortical (*N* = 40), midbrain (*N* = 9), brainstem & cerebellum (*N* = 51) (e.g., Posterior Cortex vs Posterior Cortex, *N* = 19 × 19), a list of regions is provided as a supplemental table. *=significant treatment effects, **#**=significant effects within MAR-ASD animals by sex. Effects were only reported here if they passed criteria for large effect size (Kruskal Wallis eta squared >0.14). Error bars represent the mean with 95% confidence interval.

Analysis of structural covariance in MAR-ASD and control animals, across the 159 segmented brain regions, revealed significant regional correlational differences in brain volumes by treatment and sex (Fig. 2a; Supplementary Fig. 3). To examine the nature of these changes, brain regions were first assigned to one of six anatomical clusters defined previously using unbiased hierarchical clustering of brain regions in mice [12] (Supplementary Table 2). Following assignment, correlation values within a cluster were then averaged and compared between groups using nonparametric testing. To focus on the most salient effects while considering the large number of regions within a given cluster, only those differences exhibiting a large effect size (Kruskal-Wallis eta squared >0.14) are described as significant here. However, all comparisons and relevant statistics are included as a table in the Supplementary material (Supplementary Table 5). Interestingly, when evaluating structural covariance between clusters across all conditions, male MAR-ASD mice displayed differences that were not apparent in female MAR-ASD offspring. Specifically, male MAR-ASD mice exhibited reduced structural covariance within the posterior cortex, and between the posterior and anterior cortices. Similarly, differences were also seen in intra-hippocampal connectivity, with MAR-ASD male mice displaying reduced correlations between hippocampal regions, compared to control animals of either sex as well as MAR-ASD female mice (Fig. 2b). Furthermore, structural covariance analysis revealed a number of MAR-ASD treatment-induced differences that affected male and female animals similarly. Specifically, the posterior cortex, anterior cortex, and midbrain exhibited treatment-specific reductions in their correlation to brainstem and cerebellar structures in both male and female MAR-ASD mice compared to controls (Fig. 2c). These data suggest a MAR-ASD-specific phenotype in covariance networks involving the hindbrain, irrespective of sex. Interestingly, nearly all MAR-ASD treatment-induced effects of inter- and intra-cluster correlations represented movement toward weaker or negative correlation values; suggesting desynchronized development of these regions. Cumulatively, these data propose that while regional volumetric effects were not found in male MAR-ASD mice, network-level desynchronization of structural brain volume extends to both sexes in response to MAR-ASD autoantibody exposure; providing a scaffolding for similar behavioral outcomes.

### Brain-behavior correlations reveal inverse relationships in males and females

To investigate the relationship between neuroanatomical outcomes and behavioral findings, we conducted an exploratory correlational analysis between previous behavioral findings and those brain regions determined by structural MRI to be statistically different in the same MAR-ASD mice. Bivariate correlation analysis revealed distinct sex-specific differences in both the magnitude and direction of brain-behavior correlations in respect to both JRSI (Supplementary Table 3), as well as self-grooming behaviors (Supplementary Table 4).

**Fig. 3:**
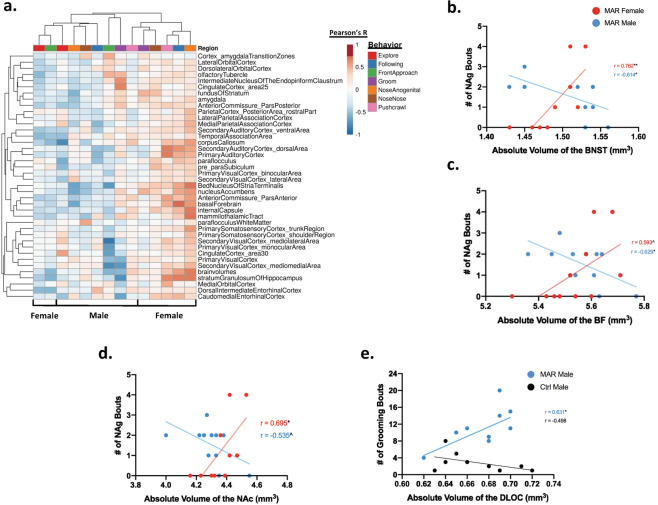
Correlational analysis between brain regions and behavior in MAR-ASD mice. **a** Clustering analysis of correlational data shown in heatmap form. Sex and behavior are displayed on the *x*-axis with brain region annotation on the *y*-axis. Clustering was conducted using Euclidean distance and average linkage analysis via the online data visualization tool ClustVis. Heatmap color for individual cells corresponds to the Pearson’s r-value, between 1 and −1, for the relationship between a given brain region and behavior in the juvenile reciprocal social interaction (JRSI) task. Correlational analysis for all data was conducted using SPSS software. **b**–**d** Scatterplot representation of correlational analysis between the number of nose-to-anogenital (NAg) bouts and volume of the BNST (MAR (N = 11M, 11F), Ctrl (N = 11M, 11F)). **b** Basal forebrain (BF) **c** and nucleus accumbens (NAc) **d** split by sex in MAR-ASD mice. **e** Graph displaying the relationship between the number of grooming bouts and volume of the dorsolateral orbital cortex (DLOC) in male MAR-ASD and control mice (MAR (N = 11M), Ctrl (*N* = 10M)). Trendlines and Pearson’s r-value displayed along with scatterplot values for each graph. * = *p* < 0.05, ** = *p* < 0.01, ^ = 0.05 < *p* < 0.1.

To further investigate the sex-specific correlational findings observed in MAR-ASD mice, we conducted clustering analysis to explore the relationship between JRSI behavior, where the majority of correlational findings were seen, and those brain regions displaying volumetric differences with an FDR < 5%. Heatmap-based visualization of clustering analysis reinforced the separation by sex in regional volumetric outcomes observed in MAR-ASD animals (Fig. 3a). While female MAR-ASD mice predominately displayed positive correlations between a given brain region and associated behavior, male MAR-ASD animals displayed either a negative correlation, or the absence of an effect across the majority of comparisons. The most striking of these sex-specific differences were seen in the relation of basal nuclei volume with nose-to-anogenital sniffing (NAg) behavior. When assessing male MAR-ASD animals, statistically significant negative correlations (here meaning correlations with a *p* value < 0.05, as no corrections for multiple comparisons were made) were seen in the BNST (*p* < 0.05, *r* = −0.614) (Fig. 3b) and basal forebrain (BF) (*p* < 0.05, *r* = −0.629) (Fig. 3c). While female MAR-ASD mice exhibited an opposing, positive relationship between NAg and regional volume in the BNST (*p* < 0.01, *r* = 0.762) and nucleus accumbens (NAc; *p* < 0.05, *r* = 0.694) (Fig. 3d). These effects appeared to be restricted to MAR-ASD animals as no statistically significant correlations were observed in control mice for either sex in regard to regional brain volume and relation to NAg (Supplementary Fig. 2b–d). In addition, visual inspection and clustering analysis of the correlational data revealed significant effects within certain brain regions with deficits spanning multiple behavioral tasks in females. Statistically significant positive correlations were observed between the volumes of the stratum granulosum of the hippocampus, caudomedial entorhinal cortex, and paraflocculus with NAg, following, and push-crawl behaviors in the JRSI tasks (Fig. 3a, Supplementary Table 3). Cumulatively these results suggest that juvenile social behavioral deficits in MAR-ASD mice appear to correlate with adult regional brain size in a sex-dependent manner. Specifically that better behavioral outcomes were associated with larger regional brain size in female MAR-ASD mice, with the opposite finding observed in male MAR-ASD offspring. However, it is important to keep in mind that these comparisons were preliminary. Future studies including additional animals or more stringent statistical analysis may be needed to validate findings.

Evaluation of additional behavioral tasks reported previously to be significantly different in mice in response to MAR-ASD exposure, such as MFSI and repetitive self-grooming behaviors, showed interesting but limited effects. As only males were tested during the MFSI task, correlations with brain volumes were conducted only with experimental male mice. In assessing MAR-ASD treatment-induced differences in male behavior during the MFSI task, one noteworthy finding included the relationship between dorsolateral orbital cortex (DLOC) volume and self-grooming behavior. Specifically, a statistically significant positive correlation was observed between the volume of the DLOC and the number of grooming bouts in male MAR-ASD mice as measured during the MFSI task (*p* < 0.05, *r* = 0.631) but not in male control mice (Fig. 3e). In addition, positive correlations were also seen between DLOC volume and total time spent grooming for male MAR-ASD mice in the MFSI task (Supplementary Fig. 2e) as well as in a separate empty-cage grooming task (Supplementary Fig. 2f). However, these last relationships did not pass statistical significance testing. Taken together, these data detail a male-specific relationship between the DLOC and repetitive self-grooming behavior in adulthood in response to MAR-ASD autoantibody exposure.

## Discussion

Abnormal brain enlargement, measured by MRI, is well documented in the ASD literature with early studies on the topic suggesting that children with ASD exhibit premature overgrowth of brain regions during early life that is followed by a period of abnormally slow growth compared to typically developing children [18–21]. However, many of these seminal reports studied mainly male children and few if any females. Later studies revealed that female children with ASD actually display a more pronounced abnormal growth profile across a greater number of brain regions than male ASD children [20, 22]. This is relevant to our findings as we observed absolute volumetric differences in white matter tracts, cerebral nuclei, cerebellum, and cerebral cortex in adult female MAR-ASD animals that were not present in males at the same time point. Therefore, it may be possible that male and female offspring experienced an accelerated neurodevelopmental trajectory during early life, which was then normalized over time in males but persisted in female MAR-ASD animals. In support of this view, increased head size was observed in both male and female MAR-ASD mice as pre-weanlings [9]. Alternatively, female-specific brain volumetric differences could reflect masculinization of the female brain in response to MAR-ASD exposure. The concept of brain masculinization in ASD has been an active theory for nearly two decades(reviewed in [23]). However, recent work suggests that sex-specific differences in brain volume in ASD may not represent extreme male skewing, but simply dysregulation of normal sexual differentiation of the brain [24]. This theory is supported by work suggesting that although females show masculinization of certain brain regions, males do not display hypermasculinization [25]. Our data appear to mirror these findings as MAR-ASD female mice show apparent masculinization of regional volumes, but the brains of male MAR-ASD mice do not appear overtly affected.

Structural covariance represents a technique to assess relationships between brain regions based on anatomical properties. Previous work has shown that this measure is related to both structural and transcriptomic similarity among regions [26], underscoring its utility. Studies using structural covariance to determine network dysfunction in ASD have evidenced altered local connectivity [27, 28], and correlations between subcortical structures that are predictive of behavioral outcomes [29]. Assessment of network connectivity using structural covariance in this study revealed reduced local covariance within the cortex and hippocampus of male MAR-ASD mice but not in females. Suggesting that while differences in discrete regional brain volume were absent in male MAR-ASD mice in this cross-sectional study, local network desynchronization may partly account for altered behavioral outcomes. Support for this exists in the literature, as cortical underconnectivity is an active hypothesis in ASD [30, 31], with several studies observing relationships between reduced functional connectivity in the cortex and worsened behavioral outcomes in affected individuals [32–34]. Furthermore, our study revealed a treatment-specific phenotype in MAR-ASD mice, irrespective of sex, noting a reduction in the covariance between cortical and midbrain regions to brainstem and cerebellar structures. Disrupted cortico-cerebellar connectivity is noted in both the clinical literature and in animal models of ASD [35, 36]. In addition, a recent study using MRI and structural covariance to examine sensory networks in individuals with ASD found evidence of decreased covariation between the cerebellum and sensory cortices [27].

Together these studies provide corroboration for the translational capacity of the MAR-ASD rodent model and reinforce the findings of this study. However, limitations exist in the cross-sectional design and the fact that the neuroimaging was conducted ex vivo. Longitudinal in vivo MRI studies will be necessary to understand the timeline and development of neuroanatomical pathology in response to MAR-ASD aAb exposure.

Structural MRI results were additionally correlated with previous behavioral findings to identify differences in neuroanatomy associated with ASD-relevant behaviors. Analysis revealed sex-specific differences in the magnitude and direction of brain-behavior correlations. Specifically, opposing relationships were observed between BF structures and social behavior in male and female MAR-ASD mice; with females displaying positive correlations while those in males were negative. Previous research has shown that inhibition of signaling in the lateral septum, a region involved in social behavior with direct inputs to the BNST, can lead to sex-dependent behavioral outcomes. Specifically, lateral septum inhibition increased juvenile social play behavior in males but decreased the same behavior in females [37]. Applying this logic to our findings, it is plausible that a volumetric increase in the BF, and a concomitant increase in local signaling, may contextualize sex differences in correlational findings between BF volume and social behavior in MAR-ASD mice. Worthy of note, lateral septal volume was larger in female MAR-ASD mice compared to control animals but did not survive FDR-correction (*q* = 0.08). While there were no statistically significant global or regional differences in brain volume seen in male MAR-ASD mice, a relationship was observed between male grooming behavior and the volume of the DLOC. This is relevant as the DLOC has been previously implicated in self-grooming behaviors using optogenetic studies in mice [38, 39]. Furthermore, the human correlate to the mouse DLOC, the dorsolateral prefrontal cortex, is thought to be involved in mediating repetitive or stereotyped behavior in clinical subjects [40, 41]. It is important to note, however, that correlational findings between regional brain volume and behavior were exploratory in nature and reflect data that were not corrected for multiple comparisons.

Speculation regarding mechanisms by which MAR-ASD autoantibodies mediate pathology may include altered developmental neuroimmune signaling. Previous work has suggested that hormones, inflammatory mediators, and the presence or absence of specific immune cells contribute to neuroanatomical sex differences in rodents. For example, neuroimaging data from T cell receptor (TCR)-deficient mice revealed that T cells were necessary for sexual dimorphism in several brain regions, including the cerebellum and BNST [42]. In addition, testosterone, endocannabinoids [43], and inflammatory molecules, such as prostaglandin E2 [44, 45], have all been implicated in brain sexual differentiation through glia-dependent signaling mechanisms. Interestingly, the window for maternal antibody transfer capable of reaching the fetal brain is defined to be between E12.5–E16.5 [46]. Neurodevelopmental events during this period include microglia colonization as well as early neurogenesis [47]. While we have not seen clear alterations to microglia in early studies of MAR-ASD embryos, we have observed autoantibody binding to radial glial cells and enhanced neurogenesis [48]. These data, alongside ongoing studies, lead us to hypothesize that MAR-ASD autoantibodies influence neuroanatomy through brain deposition and potential engagement of neuroimmune signaling pathways.

Overall, our findings suggest that MAR-ASD aAb exposure results in sex-specific changes in regional brain volume, network-level covariance among brain areas, and relationships between regional volume and ASD-relevant behaviors in a mouse model. Future studies will be necessary to establish the cellular and molecular mechanisms of MAR-ASD-induced changes in brain structure.

## Supplementary information

Supplementary Methods and Figures

Supplementary Table 1

Supplementary Table 2

Supplementary Table 3

Supplementary Table 4

Supplementary Table 5

## References

[1] APA. (2013). Diagnostic and statistical manual of mental disorders: DSM-V.

[2] Meltzer A, Van de Water J (2017). The role of the immune system in autism spectrum disorder. Neuropsychopharmacology.

[3] Ramirez-Celis A, Becker M, Nuño M, Schauer J, Aghaeepour N, Van de Water J. Risk assessment analysis for maternal autoantibody-related autism (MAR-ASD): a subtype of autism. Mol Psychiatry. 2021;26:1551–60. 10.1038/s41380-020-00998-8 PMC8159732 33483694

[4] Brimberg L, Mader S, Jeganathan V, Berlin R, Coleman TR, Gregersen PK (2016). Caspr2-reactive antibody cloned from a mother of an ASD child mediates an ASD-like phenotype in mice. Mol Psychiatry.

[5] Jones KL, Van, de Water J (2019). Maternal autoantibody related autism: mechanisms and pathways. Mol Psychiatry.

[6] Braunschweig D, Krakowiak P, Duncanson P, Boyce R, Hansen RL, Ashwood P (2013). Autism-specific maternal autoantibodies recognize critical proteins in developing brain. Transl Psychiatry.

[7] Ramirez-Celis A, Edmiston E, Schauer J, Vu T, Van, de Water J (2020). Peptides of neuron-specific enolase as potential ASD biomarkers: from discovery to epitope mapping. Brain Behav Immun.

[8] Edmiston E, Jones KL, Vu T, Ashwood P, Van de Water J (2018). Identification of the antigenic epitopes of maternal autoantibodies in autism spectrum disorders. Brain Behav Immun.

[9] Jones KL, Pride MC, Edmiston E, Yang M, Silverman JL, Crawley JN (2020). Autism-specific maternal autoantibodies produce behavioral abnormalities in an endogenous antigen-driven mouse model of autism. Mol Psychiatry.

[10] Bock NA, Nieman BJ, Bishop JB, Mark, Henkelman R (2005). In vivo multiple-mouse MRI at 7 Tesla. Magn Reson Med.

[11] Spencer Noakes TL, Henkelman RM, Nieman BJ. Partitioning k-space for cylindrical three-dimensional rapid acquisition with relaxation enhancement imaging in the mouse brain. NMR Biomed. 2017;30. 10.1002/nbm.3802 28902423

[12] Pagani M, Bifone A, Gozzi A (2016). Structural covariance networks in the mouse brain. Neuroimage.

[13] Rollins CPE, Garrison JR, Arribas M, Seyedsalehi A, Li Z, Chan RCK (2020). Evidence in cortical folding patterns for prenatal predispositions to hallucinations in schizophrenia. Transl Psychiatry.

[14] Qiu LR, Fernandes DJ, Szulc-Lerch KU, Dazai J, Nieman BJ, Turnbull DH (2018). Mouse MRI shows brain areas relatively larger in males emerge before those larger in females. Nat Commun.

[15] McCarthy MM, Wright CL (2017). Convergence of sex differences and the neuroimmune system in autism spectrum disorder. Biol Psychiatry.

[16] Mechelli A, Friston KJ, Frackowiak RS, Price CJ (2005). Structural covariance in the human cortex. J Neurosci.

[17] Mueller FS, Scarborough J, Schalbetter SM, Richetto J, Kim E, Couch A (2021). Behavioral, neuroanatomical, and molecular correlates of resilience and susceptibility to maternal immune activation. Mol Psychiatry.

[18] Nordahl CW, Braunschweig D, Iosif AM, Lee A, Rogers S, Ashwood P (2013). Maternal autoantibodies are associated with abnormal brain enlargement in a subgroup of children with autism spectrum disorder. Brain Behav Immun.

[19] Hazlett HC, Poe MD, Gerig G, Styner M, Chappell C, Smith RG (2011). Early brain overgrowth in autism associated with an increase in cortical surface area before age 2 years. Arch Gen Psychiatry.

[20] Schumann CM, Bloss CS, Barnes CC, Wideman GM, Carper RA, Akshoomoff N (2010). Longitudinal magnetic resonance imaging study of cortical development through early childhood in autism. J Neurosci.

[21] Fombonne E, Rogé B, Claverie J, Courty S, Frémolle J (1999). Microcephaly and macrocephaly in autism. J Autism Dev Disord.

[22] Bloss CS, Courchesne E (2007). MRI neuroanatomy in young girls with autism: a preliminary study. J Am Acad Child Adolesc Psychiatry.

[23] Ferri SL, Abel T, Brodkin ES (2018). Sex differences in autism spectrum disorder: a review. Curr Psychiatry Rep.

[24] Alaerts K, Swinnen SP, Wenderoth N (2016). Sex differences in autism: a resting-state fMRI investigation of functional brain connectivity in males and females. Soc Cogn Affect Neurosci.

[25] Lai MC, Lombardo MV, Suckling J, Ruigrok AN, Chakrabarti B, Ecker C (2013). Biological sex affects the neurobiology of autism. Brain.

[26] Yee Y, Fernandes DJ, French L, Ellegood J, Cahill LS, Vousden DA (2018). Structural covariance of brain region volumes is associated with both structural connectivity and transcriptomic similarity. Neuroimage.

[27] Cardon GJ, Hepburn S, Rojas DC (2017). Structural covariance of sensory networks, the cerebellum, and amygdala in autism spectrum disorder. Front Neurol.

[28] Bethlehem RAI, Romero-Garcia R, Mak E, Bullmore ET, Baron-Cohen S (2017). Structural covariance networks in children with autism or ADHD. Cereb Cortex.

[29] Duan X, Wang R, Xiao J, Li Y, Huang X, Guo X (2020). Subcortical structural covariance in young children with autism spectrum disorder. Prog Neuropsychopharmacol Biol Psychiatry.

[30] Anderson JS. Cortical underconnectivity hypothesis in autism: evidence from functional connectivity MRI. Comprehensive guide to autism. 2014, pp 1457–71.

[31] Kana RK, Libero LE, Moore MS (2011). Disrupted cortical connectivity theory as an explanatory model for autism spectrum disorders. Phys Life Rev.

[32] Rane P, Cochran D, Hodge SM, Haselgrove C, Kennedy DN, Frazier JA (2015). Connectivity in autism: a review of MRI connectivity studies. Harv Rev Psychiatry.

[33] Cheng W, Rolls ET, Gu H, Zhang J, Feng J (2015). Autism: reduced connectivity between cortical areas involved in face expression, theory of mind, and the sense of self. Brain.

[34] Khan S, Gramfort A, Shetty NR, Kitzbichler MG, Ganesan S, Moran JM (2013). Local and long-range functional connectivity is reduced in concert in autism spectrum disorders. Proc Natl Acad Sci USA.

[35] Kelly E, Meng F, Fujita H, Morgado F, Kazemi Y, Rice LC (2020). Regulation of autism-relevant behaviors by cerebellar-prefrontal cortical circuits. Nat Neurosci.

[36] Ramos TC, Balardin JB, Sato JR, Fujita A (2018). Abnormal cortico-cerebellar functional connectivity in autism spectrum disorder. Front Syst Neurosci.

[37] Bredewold R, Veenema AH (2018). Sex differences in the regulation of social and anxiety-related behaviors: insights from vasopressin and oxytocin brain systems. Curr Opin Neurobiol.

[38] Burguiere E, Monteiro P, Feng G, Graybiel AM (2013). Optogenetic stimulation of lateral orbitofronto-striatal pathway suppresses compulsive behaviors. Science.

[39] Ahmari SE, Spellman T, Douglass NL, Kheirbek MA, Simpson HB, Deisseroth K (2013). Repeated cortico-striatal stimulation generates persistent OCD-like behavior. Science.

[40] McKinnon CJ, Eggebrecht AT, Todorov A, Wolff JJ, Elison JT, Adams CM (2019). Restricted and repetitive behavior and brain functional connectivity in infants at risk for developing autism spectrum disorder. Biol Psychiatry Cogn Neurosci Neuroimaging.

[41] Kadota H, Sekiguchi H, Takeuchi S, Miyazaki M, Kohno Y, Nakajima Y (2010). The role of the dorsolateral prefrontal cortex in the inhibition of stereotyped responses. Exp Brain Res.

[42] Rilett KC, Friedel M, Ellegood J, MacKenzie RN, Lerch JP, Foster JA (2015). Loss of T cells influences sex differences in behavior and brain structure. Brain Behav Immun.

[43] VanRyzin JW, Marquardt AE, Argue KJ, Vecchiarelli HA, Ashton SE, Arambula SE (2019). Microglial phagocytosis of newborn cells is induced by endocannabinoids and sculpts sex differences in juvenile rat social play. Neuron.

[44] Lenz KM, Pickett LA, Wright CL, Davis KT, Joshi A, McCarthy MM (2018). Mast cells in the developing brain determine adult sexual behavior. J Neurosci.

[45] Wright CL, McCarthy MM (2009). Prostaglandin E2-induced masculinization of brain and behavior requires protein kinase A, AMPA/kainate, and metabotropic glutamate receptor signaling. J Neurosci.

[46] Kowal C, Athanassiou A, Chen H, Diamond B (2015). Maternal antibodies and developing blood-brain barrier. Immunol Res.

[47] Reemst K, Noctor SC, Lucassen PJ, Hol EM (2016). The indispensable roles of microglia and astrocytes during brain development. Front Hum Neurosci.

[48] Martinez-Cerdeno V, Camacho J, Fox E, Miller E, Ariza J, Kienzle D (2016). Prenatal exposure to autism-specific maternal autoantibodies alters proliferation of cortical neural precursor cells, enlarges brain, and increases neuronal size in adult animals. Cereb Cortex.

