# Supplementary material for: Sexually dimorphic neuroanatomical differences relate to ASD-relevant behavioral outcomes in a maternal autoantibody mouse model
Source: Mol Psychiatry. 2021 Jul 21;26(12):7530–7. doi: 10.1038/s41380-021-01215-w (PMC8776898; doi:10.1038/s41380-021-01215-w)
Supplement: Supplementary file 1 — Supplementary Methods and Figures [file 41380_2021_1215_MOESM1_ESM.pdf]

Supplemental Methods and Figures

Sexually dimorphic neuroanatomical differences relate to ASD-relevant behavioral outcomes in a maternal autoantibody mouse model

Matthew R. Bruce, B.S.[1], Karen L. Jones, Ph.D.[1,2], Anthony C. Vernon, Ph.D.[3,4], Jill L. Silverman, Ph.D.[2,5], Jacqueline N. Crawley, Ph.D.[2,5], Jacob Ellegood, Ph.D.[6], Jason P. Lerch, Ph.D.[6,7,8], Judy Van de Water, Ph.D.[1,2]

[1]Department of Internal Medicine, Division of Rheumatology, Allergy, and Clinical Immunology, University of California, Davis, CA, USA

[2]MIND Institute, University of California, Davis, CA, USA

[3]Department of Basic and Clinical Neuroscience, Institute of Psychiatry, Psychology and Neuroscience, King's College London, London, UK

[4]MRC Centre for Neurodevelopmental Disorders, King's College London, London UK

[5] Department of Psychiatry and Behavioral Sciences, University of California, Davis, CA, USA

[6] Mouse Imaging Centre (MICe), Hospital for Sick Children, Toronto, Ontario

[7]Department of Medical Biophysics, The University of Toronto, Toronto, Ontario

[8] Wellcome Centre for Integrative Neuroimaging, The University of Oxford, Oxford, UK.

Corresponding Author: Judy Van de Water; Division of Rheumatology/Allergy and Clinical Immunology; 451 E. Health Sciences Dr., Suite 6510; University of California Davis; Davis, CA 95616 USA. Phone: (530) 752-2154; FAX: 530-752-4669; Email: javandewater@ucdavis.edu

File Contains:

Supplemental Methods

Supplementary Fig. 1

Supplementary Fig. 2

Supplementary Fig. 3

Additional Supplementary Methods

Animals (cont.)

Briefly, all female C57BL/6J mice randomly assigned to MAR-ASD treatment received a series of immunizations containing peptide epitope sequences of the four primary target proteins of MAR ASD (LDH-A, LDH-B, STIP1, and CRMP1) and Freund's adjuvant prior to breeding. Control females were similarly injected but replacing the peptides with saline. Following confirmation of autoantibody production to the salient epitope sequences, females were then paired with male breeders to produce the experimental offspring of interest. Experimental offspring that underwent behavioral testing as juveniles and adults, as described in Jones et al, 2018, representing 1 male and 1 female from each litter (Litter Number; Control = 11, MAR-ASD = 12), were sacrificed following completion of behavioral assays, perfused, and sent to the Mouse Imaging Centre (MICe) in Toronto, ON, Canada for ex vivo magnetic resonance imaging (MRI) scanning and analysis.

Perfusions were performed at University of California, Davis prior to being shipped overnight to the Mouse Imaging Centre (MICe). At approximately 6 months of age, mice were anesthetized with isoflurane and intracardially perfused with 30 mL of 0.1 M PBS containing 10 µL/mL heparin (Sigma) and 2 mM ProHance (Bracco Diagnostics Inc., a Gadolinium contrast agent) followed by 30 mL of 4% paraformaldehyde (PFA) containing 2 mM ProHance(14). Perfusions were performed with a minipump at a rate of approximately 60 mL/hr. After perfusion, mice were decapitated and the skin, lower jaw, ears, and the cartilaginous nose tip were removed. The brain and remaining skull structures were incubated in 4% PFA + 2 mM ProHance overnight at 4°C, then transferred to 0.1 M PBS containing 2 mM ProHance and 0.02% sodium azide for at least one month prior to MRI scanning(15).

Registration and Analysis

To visualize and compare potential volume changes in the mouse brains, the MR images were linearly (6 parameter followed by a 12 parameter) and non-linearly registered together. All scans were then resampled with the appropriate transform and averaged to create a population atlas representing the average anatomy of the entire

study sample. The result is aligned scan deformation in an unbiased fashion. This allows for the analysis needed to take each individual mouse's anatomy into this final atlas space. The log-transformed Jacobian determinants of the deformation fields were then calculated as measures of volume at each voxel. Significant volume changes are then calculated by warping a pre-existing classified MRI atlas onto the population atlas, which allows for the volume of 159 segmented structures encompassing cortical lobes, large white matter structures (i.e. corpus callosum), ventricles, cerebellum, brain stem, and olfactory bulbs to be assessed in all brains. Multiple comparisons in this study were controlled for using the False Discovery Rate (FDR). To assess whether findings varied across biological sex, three sets of analyses were completed for each region: 1) "Full" group comprised of both males and females, 2) males only, and 3) females only.

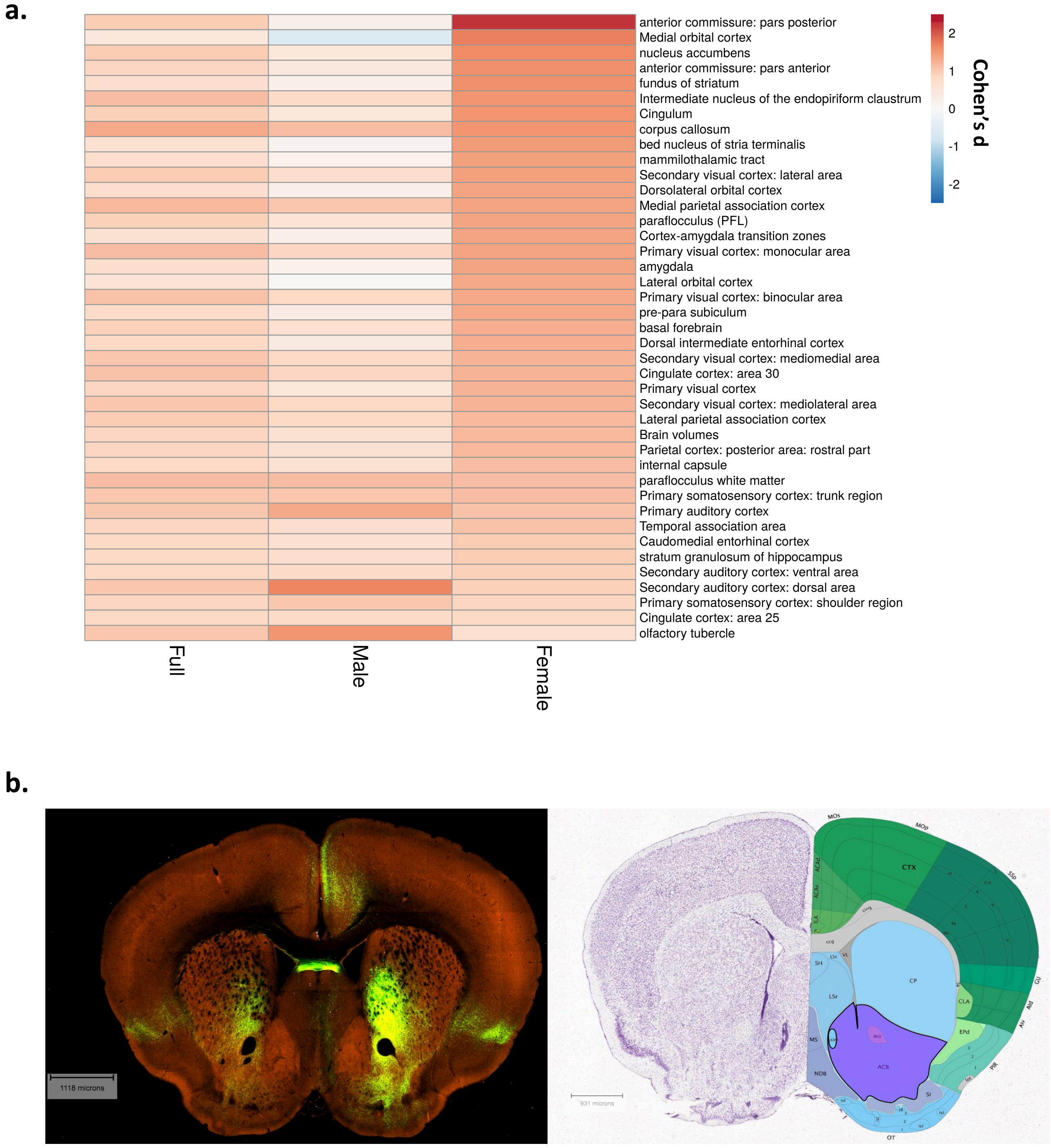

Supplementary Figure 1. Effect sizes of volumetric changes in MAR-ASD female mice implicate altered cortico-striatal connectivity. a) Heatmap plot displaying effect sizes by region for male and female MAR-ASD mice as well as combined (Full). Effect sizes represent Cohen's d values. Legend for effect sizes has a range between 2.5 & -2.5 (MAR (N = 11M, 11F), Ctrl (N = 12M, 11F)). **b)** Images collected from the Allen Mouse Brain Connectivity and Reference Atlas's. The first image details labeling in response to fluorescent tracer injection into the mOFC of wildtype C57Bl/6 mice. The second image is for reference of labeled regions. Highlighted in purple is the nucleus accumbens and anterior commissure pars anterior. Fluorescent staining also appears to a large extent in the caudoputamen.

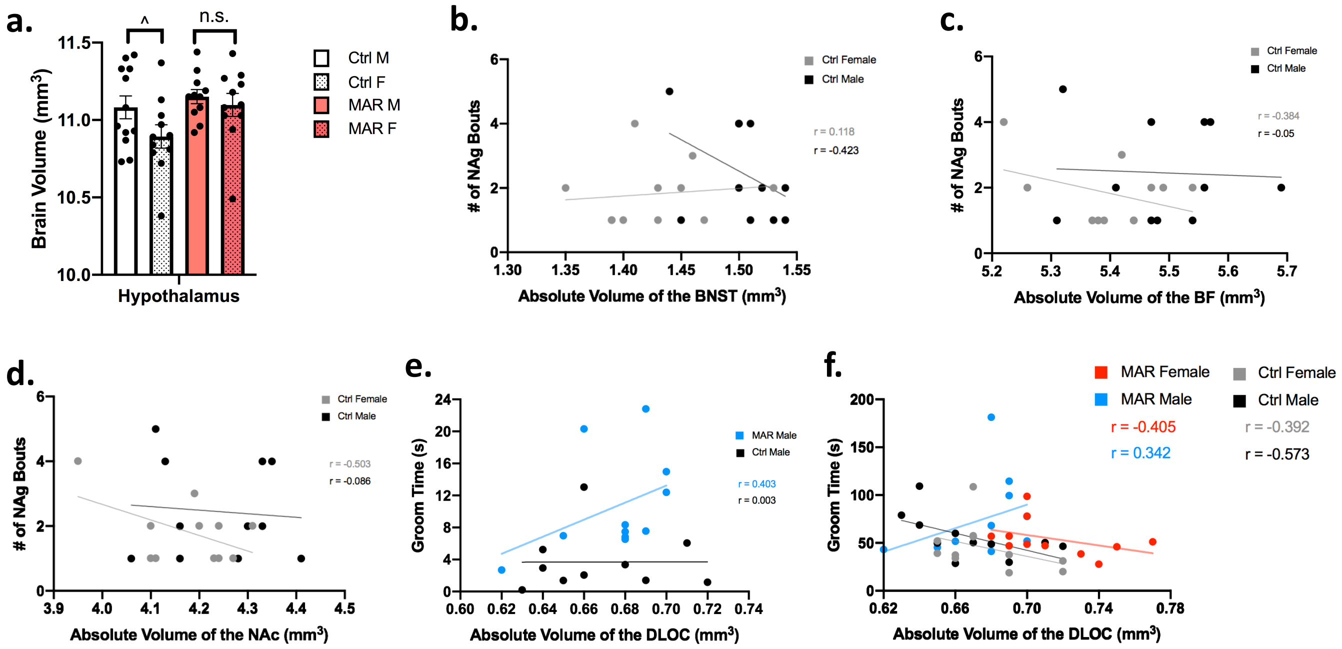

Supplementary Figure 2. Graphical representation of additional volumetric brain data and correlational findings. a) Brain volume of the hypothalamus analyzed between sexes within treatment. Statistical analysis conducted using a one-way ANOVA (MAR (N = 11M, 11F), Ctrl (N = 12M, 11F)). Error bars represent the mean +/- SEM. ^ = $0.05<p<0.1$, n.s. = non-significant. **b-d)** Correlation between the number of nose-to-anogential (NAg) bouts during the JRSI task and volume of the BNST (MAR (N = 11M, 11F), Ctrl (N = 11M, 11F)). **(b),** basal forebrain (BF) **(c)**, and nucleus accumbens (NAc) **(d)** in control animals, split by sex. **e)** Relationship between groom time (seconds) and volume of the dorsolateral orbital cortex (DLOC) in male mice during the male female social interaction (MFSI) task (MAR (N = 11M), Ctrl (N = 10M)). **f)** Graph of correlations across treatment and sex for groom time (seconds), as measured during empty cage grooming behavior, and volume of the DLOC (MAR (N = 11M, 11F), Ctrl (N = 11M, 11F)). Trendlines and Pearson's r-value displayed along with scatterplot values for each graph**.**

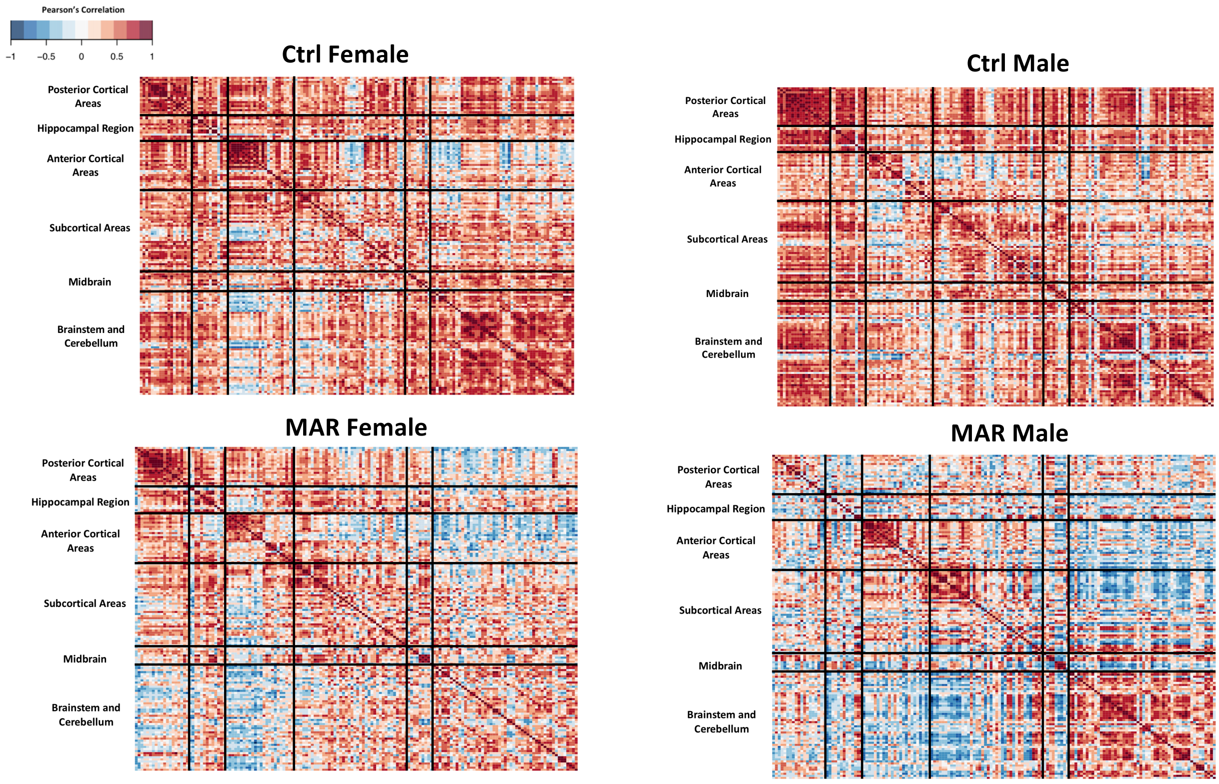

Supplementary Figure 3. Heatmap plots of the structural covariance data before averaging within clustered regions. Data represent correlational comparisons between all 159 regions split by treatment and sex. Clusters are demarcated by black lines. Heatmap legend represents Pearson's r values between a range of 1 to -1.
